# A Concise Review of the Multimodality Imaging Features of Renal Cell Carcinoma

**DOI:** 10.7759/cureus.13231

**Published:** 2021-02-08

**Authors:** Ali Morshid, Elif S Duran, Woongsoon J Choi, Cihan Duran

**Affiliations:** 1 Diagnostic Radiology, The University of Texas Medical Branch at Galveston, Galveston, USA; 2 Diagnostic Radiology, University of Texas Rio Grande Valley School of Medicine (UTRGV) School of Medicine, Edinburg, USA; 3 Radiology, Mcgovern Medical School at Uthealth, Houston, USA

**Keywords:** rcc, mri, ct

## Abstract

The evaluation of renal cell carcinoma (RCC) is routinely performed using the multimodality imaging approach, including ultrasonography, computed tomography (CT), magnetic resonance imaging (MRI), and positron emission tomography (PET). Ultrasonography is the most frequently used imaging modality for the initial diagnosis of renal masses. The modality of choice for the characterization of the renal mass is multiphasic CT. Recent advances in CT technology have led to its widespread use as a powerful tool for preoperative planning, reducing the need for catheter angiography for the evaluation of vascular invasion. CT is also the standard imaging modality for staging and follow-up. MRI serves as a problem-solving tool in selected cases of undefined renal lesions. Newer MRI techniques, such as arterial spin labeling and diffusion-weighted imaging, have the potential to characterize renal lesions without contrast media, but these techniques warrant further investigation. PET may be a useful tool for evaluating patients with suspected metastatic disease, but it has modest sensitivity in the diagnosis and staging of RCC. The newer radiotracers may increase the accuracy of PET for RCC diagnosis and staging. In summary, the main imaging modality used for the characterization, staging, and surveillance of RCC is multiphasic CT. Other imaging modalities, such as MRI and PET, are used for selected indications.

## Introduction and background

Renal cell carcinoma (RCC) constitutes 85% to 90% of all renal malignancies and represents 3% of all cancers in adults. According to 2019 cancer statistics data, RCC is the seventh and eighth most common cancer in males and females, respectively, in the United States [1]. Fifty percent to 60% of RCCs are discovered incidentally with increased incidence over the past few decades [2].

RCC represents a family of related tumors that differ in histopathology, prognosis, and imaging characteristics. It can be familial or sporadic. Endogenous risk factors for RCC include older age, male sex, and inherited genetic syndromes, including von Hippel-Lindau disease, hereditary papillary renal cancer, and tuberous sclerosis. Exogenous risk factors for developing sporadic RCC include cigarette smoking, obesity, dietary factors, hypertension, taking antihypertensive medications or analgesics, hormonal and reproductive factors, and radiation exposure [3].

The von Hippel Lindau (VHL) tumor suppressor gene is inactivated in most clear cell RCCs [4]. The defective VHL tumor suppressor protein is unable to degrade hypoxia-inducing factors (HIF), which results in the upregulation of vascular endothelial growth factor (VEGF) and platelet-derived growth factor (PDGF). One of several related metabolic pathways, the mammalian target of rapamycin (mTOR) pathway is also activated. These growth factors and pathways promote tumor cell growth, survival, and angiogenesis and are responsible for the hypervascularity of clear cell carcinoma [5].

According to the 2016 World Health Organization (WHO) classification system, the histological subtypes of RCC are clear cell carcinoma (ccRCC, also called “conventional RCC”), multilocular cystic renal neoplasm of low malignant potential, papillary RCC, chromophobe RCC, collecting duct carcinoma, MT family translocation RCC, mucinous tubular and spindle cell carcinoma, and unclassified type RCC [6]. Sarcomatoid variants can occur with any of the subtypes. The histopathologic subtype affects the tumor’s biologic behavior, the patient’s prognosis, and the choice of treatment.

In this article, we will review imaging techniques used in the evaluation of RCC, including computed tomography (CT), magnetic resonance imaging (MRI), ultrasonography, and positron emission tomography (PET) [7].

## Review

The role of imaging

The main goal of imaging in the management of RCC is the characterization of the renal mass or masses. CT, MRI, ultrasonography, and PET are the imaging modalities used for the characterization of RCC as well as its diagnosis, staging, and follow-up.

The first step in characterizing RCC is to determine whether the lesion appears cystic or solid on imaging. Cystic renal lesions usually are classified on the basis of their CT appearance using the Bosniak renal cyst classification system, which can help determine the malignancy risk and follow-up requirements [8-9]. There was a recent proposal for an update to the system with more clearly defined imaging terms and incorporation of MRI. The system assigns cystic lesions to one of five categories on the basis of CT and MRI findings such as the presence of septa, calcifications, solid nodular components, or enhancing soft tissue components; thickening or measurable enhancement of the septa or wall; and the attenuation value of the lesion (Table 1).

**Table 1:** Proposed Bosniak renal cyst classification system 2019 Adapted from Silverman SG, Pedrosa I, Ellis JH, et al. (2019) Bosniak classification of cystic renal masses, version 2019: an update proposal and needs assessment. Radiology 292:475–488 [8]

Category	Characteristics
I	Benign, simple cyst with a well-defined, thin smooth wall (≤2 mm). No septa, calcifications, or solid components. Attenuation equivalent to simple fluid (-9 to 20 HU); the wall may enhance.
II	Six types, all with thin smooth walls: Cystic masses demonstrating few septa that may enhance. May also have any types of calcifications. Homogeneous hyperattenuating (≥70 HU) masses at unenhanced CT. Homogeneous nonenhancing masses (>20 HU) at renal mass protocol CT. May also have any type of calcifications. Homogeneous masses -9 to 20 HU at unenhanced CT. Homogeneous masses 21 to 30 HU at portal venous phase CT. Homogeneous low attenuation masses that are too small to characterize.
IIF	Cysts with minimally thickened (3 mm) smooth enhancing wall or septa or many (≥ 4) smooth thin (≤2 mm) enhancing septa.
III	Cystic mass with thickened irregular (≥4 mm width) or smooth walls or septa in which measurable enhancement is present.
IV	Clearly malignant cystic mass that not only meets all the criteria of category III but also contains enhancing soft tissue components (≥4 mm) independent of the wall or septa.

While category I and most category II lesions are accepted as benign and do not require further intervention, category IIF lesions require follow-up. Surgery is indicated for category III lesions, which have a substantial malignancy risk, and for category IV lesions, which are clearly malignant.

In the evaluation of solid RCCs, contrast enhancement on CT and MRI are the most reliable indication that a renal lesion is a neoplasm. Imaging also plays an important role in the staging of RCC. Accurate staging is essential for preoperative planning, especially when nephron-sparing surgery is being considered. The other purposes of imaging in RCC management are follow-up after surgery and other interventions and assessment of the response to targeted therapy in patients with advanced RCC.

Evaluation by CT

CT is the main imaging modality used for the evaluation of renal tumors in most institutions and can detect RCC with a sensitivity of 95% to 100% and specificity of 88% to 95% [10]. RCC may appear on CT as a solid or a cystic mass secondary to hemorrhage or necrotic degeneration. Central or peripheral calcification occurs in 20% of RCCs, and on rare occasions, macroscopic fat can be observed in the tumor [11]. Among patients with RCC, 25% to 30% have metastases at presentation [1].

The use of a dedicated renal CT protocol is necessary for the comprehensive evaluation of renal masses. The renal CT protocol consists of the following phases: precontrast, corticomedullary (40-60 seconds delay), nephrographic (80-90 seconds delay), and excretory (180-300 seconds delay). Pre-contrast images detect calcification, hemorrhage, and fat and provide a baseline for the quantification of enhancement on the postcontrast images [12]. Corticomedullary-phase images are useful for differentiating clear cell RCC from the other subtypes and provide valuable information about the renal vascular anatomy. In the nephrographic phase, the renal parenchyma enhances homogeneously, maximizing the opportunity for the detection and characterization of hypovascular renal masses. Excretory-phase images define the relationship of the tumor to the collecting system, which is important in planning nephron-sparing surgery.

**Figure 1:**
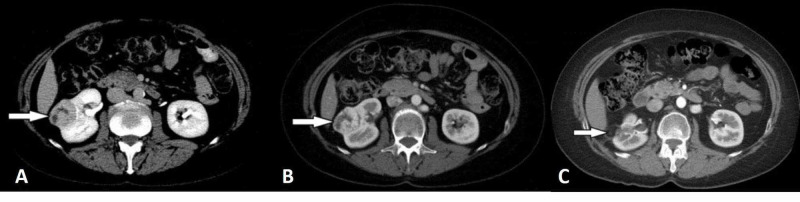
Clear cell renal cell carcinoma (arrows) Computed tomography images taken in the corticomedullary (a) and excretory (b) phase images show typical hypervascularity and subsequent washout of the tumor. In the same patient after nephron-sparing surgery (c), no residual or recurrent tumor is detected.

Clear cell RCCs are typically hypervascular and enhance to a similar degree as the renal cortex in corticomedullary-phase images (Figure 1).

**Figure 2:**
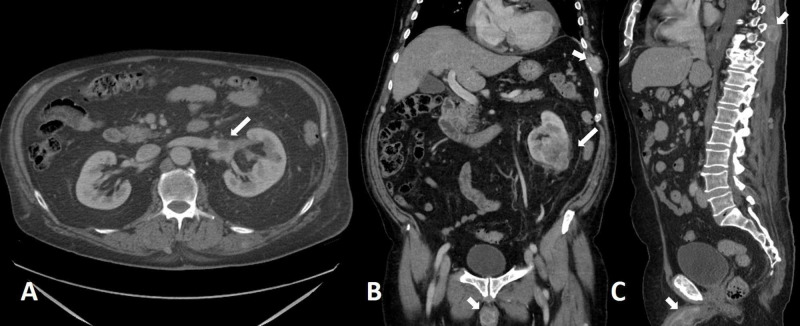
Clear cell renal cell carcinoma (long arrows) Computed tomography images taken in the axial (a), coronal (b), and sagittal (c) planes show a large heterogeneously enhancing left lower renal pole lesion with extension in the left renal vein (axial, long arrow). Sagittal and coronal reformats demonstrate penile and musculoskeletal metastatic disease (short arrows).

Kim and colleagues showed that an increase in attenuation of 84 Hounsfield units in corticomedullary-phase scans differentiated clear cell RCCs from non-clear cell tumors with a sensitivity of 74% and a specificity of 100% [13]. Rupper-Kohlmayer et al. reported the use of 100 HU as a cutoff for differentiation of ccRCC from papillary RCC with an accuracy of 95.7%, and sensitivity and specificity of 98.3% and 92%, respectively [14]. If a lesion enhances less than the renal cortex in corticomedullary-phase images, the lesion is most likely a non-clear cell subtype of RCC. Extension of the tumor into the renal vein and vena cava is a well-recognized feature of RCC [15]. Hötker et al. examined a cohort of 763 patients for the association between CT findings and patient survival. They found that tumor size, renal vein invasion, and the presence of extensive necrosis were associated with decreased survival (Figure 2) [16].

**Figure 3:**
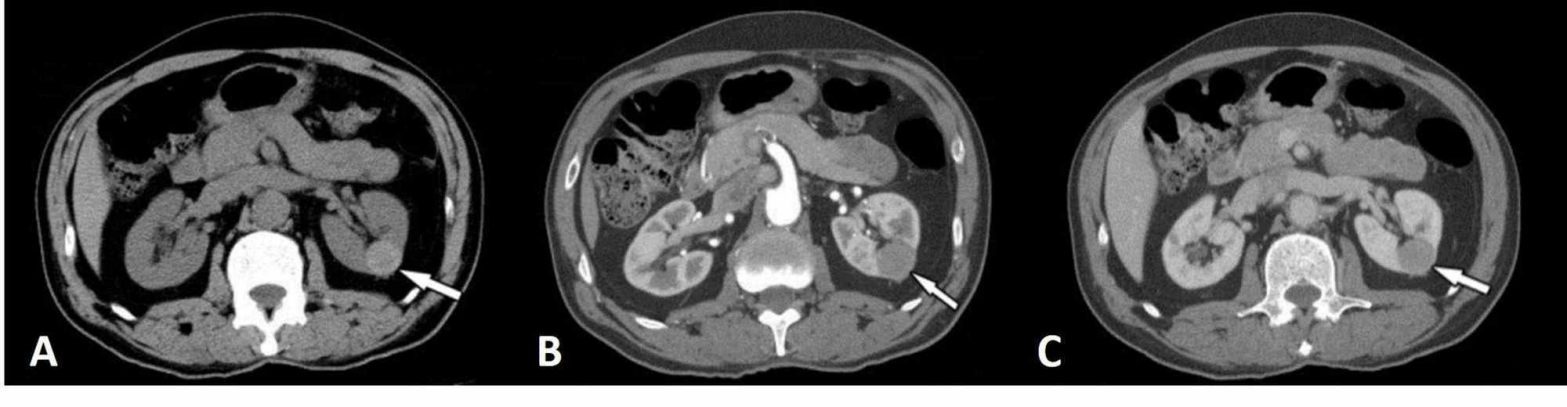
Papillary renal cell carcinoma (arrows) Unenhanced (a), corticomedullary-phase (b), and nephrographic-phase (c) computed tomography images show a precontrast homogeneous hyperattenuating, hypovascular mass lesion without significant enhancement in the left kidney.

Papillary RCCs often are homogeneous and hypoenhancing as compared with the renal parenchyma. Papillary RCCs also show less enhancement than clear cell RCCs in all postcontrast phases [2] (Figure 3).

Papillary RCCs smaller than 3 cm in diameter tend to be more homogeneous than larger papillary tumors [17]. Large papillary RCCs can be more necrotic and heterogeneous, but the tumor nodules enhance less than the renal cortex in corticomedullary-phase images. Papillary RCCs can be hyperdense in pre-contrast images and may look like pseudocystic lesions. There are two morphologic subtypes of papillary RCC, and it is not possible to differentiate type I from type II by CT [2].

Chromophobe RCCs are less hypovascular and tend to be more homogeneous than papillary RCCs, even when they are large [12]. Their enhancement characteristics are variable and fall in between ccRCC and papillary RCC. Chromophobe RCCs can grow to a large size without undergoing central necrosis; however, they often demonstrate a central scar [18].

Renal medullary carcinomas demonstrate limited postcontrast enhancement. Most renal medullary carcinomas are poorly differentiated, infiltrative, and indistinguishable from collecting duct tumors on CT; however, they occur mostly in young patients with sickle cell trait. Most collecting duct carcinomas are heterogeneous, hypodense masses that can be highly aggressive.

Although clear cell RCCs have characteristic imaging features, the various types of non-clear cell RCCs cannot be easily differentiated from each other by imaging findings, and tissue sampling or surgical resection is required for the definitive diagnosis of non-clear cell RCCs in most cases [19].

Renal oncocytomas are typically hypervascular on contrast-enhanced CT. Differentiation between renal oncocytoma and RCC by imaging is not reliable even if the oncocytoma has a prominent central scar, as a central scar can also be present in necrotic clear cell RCC [19-20].

Angiomyolipoma often can be differentiated from RCC by identifying macroscopic fat within a solid mass, which is typically seen with angiomyolipoma. This differentiation may be difficult in cases such as angiomyolipoma with microscopic fat or large RCCs, which may contain a small amount of fat [21].

For RCC staging purposes, CT has a reported accuracy rate of 91%. RCC staging includes the assessment of tumor size and location, invasion of the renal sinus and calyces, invasion of the ipsilateral renal vein, extension into the inferior vena cava, direct extension into adjacent organs, the involvement of lymph nodes, ipsilateral or contralateral adrenal involvement, and distant metastatic disease (Table 2) [22].

**Table 2:** TNM classification of renal cell carcinoma TNM: tumor, node, metastases Adapted from Edge SB, Bird DR, Compton CC, Fritz AG, Greene FL, Trotti A, eds. AJCC Cancer Staging Handbook. 7th ed. New York: Springer; 2010 [22]

Primary tumor	
TX	Primary tumor cannot be assessed
T0	No evidence of primary tumor
T1	Tumor confined to the kidney ≤ 7 cm in the greatest dimension
T1a	Tumor confined to the kidney ≤ 4 cm in the greatest dimension
T1b	Tumor confined to the kidney >4 cm but ≤7 cm in the greatest dimension
T2	Tumor confined to the kidney >7 cm in the greatest dimension
T2a	Tumor confined to the kidney >7 cm but ≤10 cm in the greatest dimension
T2b	Tumor confined to the kidney >10 cm
T3	Tumor extends into major veins or perinephric tissues but not into the ipsilateral adrenal gland and not beyond the Gerota fascia
T3a	Tumor grossly extends into the renal vein or its segmental branches, or tumor invades perirenal and/or renal sinus but not beyond the Gerota fascia
T3b	Tumor grossly extends into the vena cava below the diaphragm
T3c	Tumor grossly extends into the vena cava above the diaphragm or invades the wall of the vena cava
T4	Tumor invades beyond the Gerota fascia (including contiguous extension into the ipsilateral adrenal gland)
Regional lymph nodes	
NX	Regional lymph nodes cannot be assessed
N0	No regional lymph node metastasis
N1	Metastasis in regional lymph node(s)
Distant metastasis	
M0	No distant metastasis
M1	Distant metastasis

Most RCC staging errors are related to the evaluation of the extracapsular tumor extension [23]. The detection of perinephric invasion, renal sinus fat invasion, and calyceal involvement are particularly important in surgical planning.

Three-dimensional (3D) volume rendering, multiplanar reformatting, and maximum intensity projection facilitate preoperative planning by visualizing the relationships of the renal structures and help the surgeons in preoperative planning. 3D-CT images have reduced the need for diagnostic catheter angiography in preoperative planning, but the procedure remains indicated for therapeutic interventional measures [24].

Evaluation by MRI

MRI, with its superior soft-tissue contrast, has a sensitivity and specificity comparable to that of CT for the detection and characterization of small renal masses [25], but MRI is less accessible, more expensive, and requires a longer examination time.

Renal MRI protocols include non-contrast T1- and T2-weighted sequences, chemical shift imaging, which permits the detection of fat, and dynamic contrast-enhanced 3D gradient-echo sequences, which allow the evaluation of tumoral contrast enhancement. Multiple dynamic acquisitions are typically used to obtain corticomedullary-, nephrographic-, and excretory-phase images. A coronal 3D fast-gradient echo sequence with fat suppression after a dynamic series is useful for evaluating the renal venous anatomy and inferior vena cava for tumor thrombus. The ability to subtract post- and pre-contrast images obtained with 3D gradient-echo sequences can help the detection of subtle enhancement [26].

Clear cell RCCs usually appear isointense or hypointense relative to renal parenchyma on precontrast T1-weighted images and heterogeneously hyperintense on T2-weighted images. As on CT, clear cell RCCs are hypervascular, show prominent enhancement on MRI, especially in the corticomedullary phase, unlike other RCC subtypes [27]. Clear cell RCCs also demonstrate rapid washout of contrast with T2 hypointensity on the excretory phase. Chemical shift imaging can be useful in identifying clear cell RCC by detecting the presence of microscopic fat. The presence of necrosis or hemorrhage may alter the signal intensity characteristics of the tumor [27]. Doshi et al. studied preoperative MRI features in 230 RCCs and demonstrated that cystic and T1-hypointense RCCs are associated with a less aggressive and more favorable clinical course [28].

**Figure 4:**
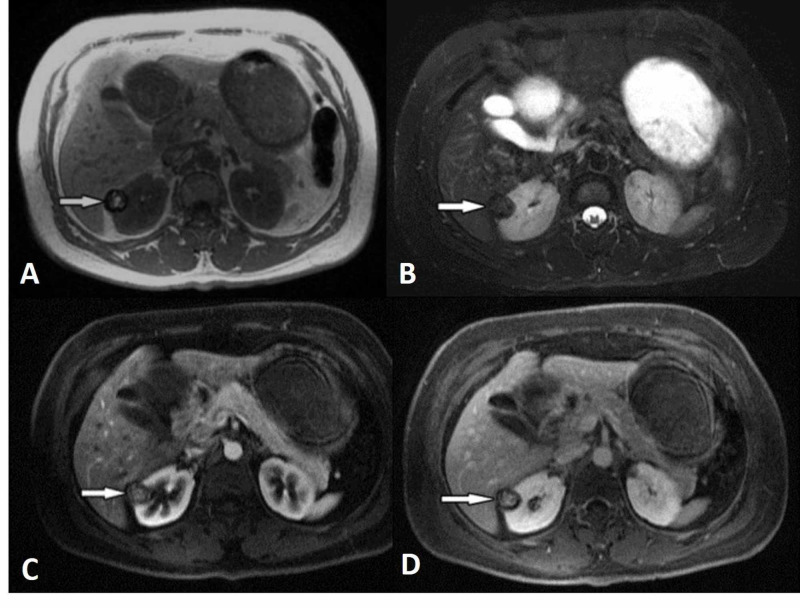
Papillary renal cell carcinoma (arrows) Unenhanced axial T1-weighted (a), fat-saturated T2-weighted (b), and contrast-enhanced T1-weighted (c, d) magnetic resonance images show a well-demarcated, hypovascular cortical lesion with a hemosiderin rim in T1-weighted images in the right kidney.

Papillary RCCs show homogeneously low signal intensity on T2-weighted MRI sequences because of intratumoral hemosiderin and low levels of enhancement in contrast-enhanced dynamic series (Figure 4).

Sun and colleagues found that clear cell RCCs had greater signal intensity change than did papillary RCCs on both corticomedullary- and nephrographic-phase images and that chromophobe RCCs showed an intermediate signal intensity change. The researchers found that signal intensity changes on corticomedullary-phase images were the most effective parameter for distinguishing between clear cell and papillary RCC [29].

The overall accuracy of MRI in RCC staging is similar to that of multidetector CT [27]. MRI is superior to earlier generation CT for venous thrombus diagnosis and staging, but newer CT technology is considered comparable to MRI for accurately showing venous thrombosis. Several studies have shown that quantitative MRI techniques such as arterial spin labeling (ASL), diffusion-weighted imaging (DWI), and intravoxel incoherent motion (IVIM) have the potential for assessing tumor vascularization. This is useful in differentiating between RCC subgroups and determining response to therapy [30-31]. MRI is superior to CT, as it avoids ionizing radiation and can detect very small enhancing elements within complex cystic masses [32].

Evaluation by ultrasonography

Most incidental renal masses are identified on ultrasonography. The main role of ultrasonography in the diagnosis of renal masses is distinguishing solid masses from simple cysts. Ultrasonography has not demonstrated utility in differentiating among the subtypes of RCC. Although Doppler ultrasonography can detect vascularization in a solid lesion, CT or MRI with a dedicated renal protocol is necessary for further evaluation. For these reasons, ultrasonography has been replaced by CT and MRI as a diagnostic and staging tool for renal masses.

Nevertheless, intraoperative ultrasonography is very useful for guiding partial nephrectomy. An accurate depiction of the relationship of the tumor to the vascular structures and calyces enables the surgeon to avoid injury to these structures and achieve negative resection margins [33]. Intraoperative ultrasonography is also used to guide laparoscopic radiofrequency ablation or cryotherapy of renal tumors.

Contrast-enhanced ultrasound (CEUS) is a relatively inexpensive and harmless modality that has comparable diagnostic confidence to CT and MRI in differentiation between benign and malignant renal lesions [34]. However, CEUS's role is limited given the lack of anatomic detail evaluation and its operator dependence. It can, however, be used as a supplemental tool in conjunction with contrast-enhanced CT.

Evaluation by PET

Because fluorodeoxyglucose (FDG) uptake by RCCs is limited, the role of FDG-PET for the initial detection and diagnosis of RCC is minor. Published studies report a broad range of accuracy rates for FDG-PET in diagnosis and staging in patients with RCC [35]. However, FDG-PET has some potential for the detection of local recurrence and can be used as a complementary tool to other cross-sectional imaging techniques. Also, FDG-PET may be useful in the evaluation of tumor response to targeted therapy with tyrosine kinase inhibitors [36-37]. The value of PET radiotracers other than FDG in the evaluation and management remains a topic of ongoing research [38].

Evaluation by SPECT

Single-photon emission computed tomography (SPECT) using technetium (99mTc) sestamibi is a molecular imaging modality that can help differentiate between benign and malignant renal masses. This test is based on normal renal parenchymal uptake of the radiotracer, which allows the differentiation between oncocytoma and RCC. Oncocytoma will demonstrate increased radiotracer uptake similar to the surrounding parenchyma while RCC will have lower uptake relative to the surrounding parenchyma [39].

Follow-up evaluation

Radical nephrectomy, nephron-sparing surgery, and minimally invasive techniques, such as radiofrequency ablation or cryoablation, are the treatment options for RCC, and their use is determined by the stage of the tumor. For metastatic RCC, immunotherapy with interleukin-2 and interferon-α has been largely replaced by tyrosine kinase inhibitors, such as VEGF inhibitors and mTOR inhibitors [40- 41]. Imaging studies are used for surveillance following nephrectomy and percutaneous ablation to evaluate residual, recurrent, or metastatic disease and to monitor the response to targeted or systemic therapy.

Local recurrence of RCC in the tumor bed occurs in 20% to 40% of patients in the first five years after nephrectomy [1,42]. The most frequent metastatic sites are the lungs, bones, lymph nodes, and liver; the less frequent metastatic sites are the ipsilateral adrenal gland, pancreas, contralateral kidney, brain, and retroperitoneum. Risk-adapted approaches are usually used for the evaluation of possible metastatic sites [43].

CT or MRI is used to monitor abdominal metastases. Chest radiography or chest CT may be preferred for the evaluation of lung metastasis. Brain MRI or a nuclear medicine bone scan is required only when there are symptoms suggestive of metastasis to brain and bone, respectively.

Ziho et al. proposed a novel classification system for the local recurrence of RCC following surgical resection. They classified local recurrence as type I-single recurrence in a residual kidney or ipsilateral surgical bed, type II-single recurrence in the ipsilateral vascular structures, adrenal gland or a lymph node, type III-single recurrence in different intra-abdominal organs, and type IV-any combination of types I-III or multiple single-type recurrences [44]. They demonstrated that types I-III single recurrence have a similar prognosis with no difference in overall survival. Type IV local recurrence demonstrated worse five-year cancer-specific survival and overall survival.

**Figure 5:**
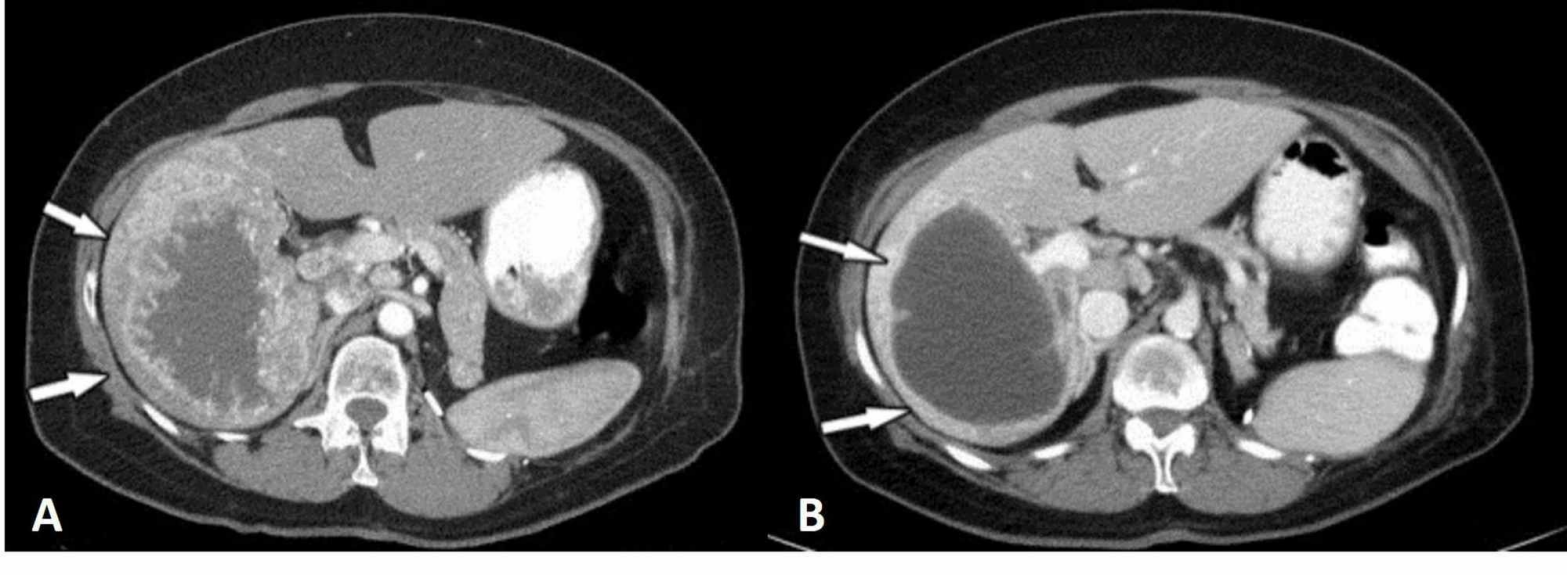
Liver metastasis from renal cell carcinoma (arrows) before (a) and after (b) VEGF inhibitor therapy The lesion has decreased in size only slightly but shows a prominent attenuation decrease after therapy.

Until recently, the evaluation of therapeutic response in metastatic RCC was mostly based on the Response Evaluation Criteria in Solid Tumors (RECIST) but these criteria are based on changes in tumor size and may not adequately reflect the response to targeted therapies, which mainly induce tumor necrosis with little tumor shrinkage (Figure 5) [45].

New response evaluation criteria that include size, as well as treatment-induced morphologic changes to the tumor, have been under development for the evaluation of therapeutic response [46-48].

Technical improvements to imaging have allowed the physiologic and functional assessments of RCCs, including the evaluation of tumor perfusion and diffusion characteristics. It has been shown that tumors with high blood volume and high blood flow are more likely to respond to antiangiogenic therapy; thus, renal carcinoma perfusion parameters can help predict and detect the response of metastatic RCC to antiangiogenic therapy [49-50]. Although functional imaging techniques are not widely available for clinical use at present, it is predicted that they will play an important role in the evaluation of metastatic RCC response to treatment.

## Conclusions

The imaging of RCC is complex and utilizes different modalities and techniques. Ultrasonography is the most frequently used modality for the initial diagnosis of renal masses. CT is the main imaging modality used for the characterization, staging, and surveillance of RCC. Recent advances in CT technology have led to its widespread use as a powerful tool for preoperative planning. MRI is mainly used as a problem-solving tool in selected cases of undefined renal lesions. Familiarity with RCC imaging characteristics on these modalities is essential to reach an accurate diagnosis and avoid misinterpretation.
